# Rotavirus Serotype G9P[8] and Acute Gastroenteritis Outbreak in Children, Northern Australia

**DOI:** 10.3201/eid1009.040040

**Published:** 2004-09

**Authors:** Carl Kirkwood, Nada Bogdanovic-Sakran, Graeme Barnes, Ruth Bishop

**Affiliations:** * Murdoch Childrens Research Institute, Parkville, Victoria, Australia;; † Royal Children's Hospital, Victoria, Australia;; ‡ University of Melbourne, Victoria, Australia

**Keywords:** rotavirus, acute gastroenteritis, serotype G9, Australia, research

## Abstract

During 2001, an outbreak of severe acute gastroenteritis swept through Central and northern Australia and caused serious disruption to health services. We tracked and characterized the rotavirus strain implicated in the outbreak. Comparison of the electropherotypes of outbreak samples suggested that one G9P[8] strain was likely responsible for the outbreak. Samples were obtained from geographically distinct regions of Australia where the epidemic had occurred. The outbreak strains showed identical nucleotide sequences in genes encoding three rotavirus proteins, VP7, VP8, and NSP4, but they were distinct from G9P[8] strains isolated in previous years. Several of the amino acid substitutions on the VP7 and NSP4 proteins were identified in regions known to influence function and may have contributed to the emergence and increased dominance of the outbreak strains. Rotavirus serotype surveillance should continue with methods capable of identifying new and emerging types.

## Abstract 2

Amino acid substitutions on the VP7 and NSP4 proteins were identified in regions known to influence function and may have contributed to the emergence and increased dominance of the outbreak strains.

Rotaviruses are the major cause of severe gastroenteritis in young children worldwide. Surveillance studies and serum antibody studies indicate that all young children are likely to have had at least one rotavirus infection by the time they are 3 years of age. Worldwide, approximately 400,000–600,000 children in developing countries die of rotavirus-associated dehydration each year (*1*). Most deaths occur in developing countries because of delays in access to treatment. Despite low death rates in industrialized countries, good hygiene and sanitation do not appear to reduce the prevalence or prevent the spread of rotavirus.

Since 1983, vaccines to protect against clinically severe disease have been under development. The first vaccines were aimed at providing specific protection against the serotypes G1, G2, G3, and G4, which were predominant since 1973 (*2*). Rotavirus surveillance programs in Bangladesh (*3*), Brazil (*4*), India (*5*), the United States (*6*), and Malawi (*7*) show that additional G types (G5, G6, G8, G9, G10) can cause severe disease in children and are of emerging importance in some communities. Serotype G9 is recognized as the most widespread of the emerging serotypes and is now considered the fifth major G type. The serotype has been identified since 1996 as a frequent cause of severe disease in hospitalized children from many countries, including the United States, Japan, India, Bangladesh, France, Malawi, Nigeria, Australia, China, Thailand, and the United Kingdom (*3**,**6**,**8**–**12*).

Characterizing rotaviruses into serotypes is based on differences in genetic and antigenic structure of the two outer coat proteins. The rotavirus genome is made up of 11 segments of double-stranded RNA located inside the core of a triple-layered structure. The outer capsid proteins, VP4 and VP7, elicit neutralizing antibody immune responses that are both serotype-specific and cross-reactive (*13*). Antigenic differences in VP4 and VP7 are the basis of the classification into G (VP7 glycoprotein) and P (protease-activated VP4 protein) serotypes. To date, 8 P serotypes and 10 G serotypes have been identified in humans by cross-neutralization tests (*13**,**14*). Epidemiologic studies have shown that serotypes G1, G2, G3, and G4, associated with P1A[8] or P1B[4], have been the most common serotypes to cause severe disease in children worldwide in the 1980s and 1990s (*2*). Genetic and antigenic variation has been recorded within G1, G2, G3, and G4 serotypes (*15*). G9 strains may be more susceptible to genetic change than these other serotypes (*3**,**16*).

The emergence of G9 strains in urban Australia in 1997, together with the increasing prevalence and persistence of this serotype, has had a major effect on healthcare services in Australia (*11*). G9 strains were initially identified in Central Australia in 1999 in 9% of children admitted to the Alice Springs Hospital but appeared not to persist, since the strains were not detected in this area in 2000 (*17*). During May 2001, one of the largest recorded outbreaks of severe acute gastroenteritis in young aboriginal children from remote and urban areas of Central Australia resulted in 246 emergency department visits and the hospitalization of 137 children at Alice Springs Hospital (*18*). All specimens from hospitalized children were rotavirus positive. The epidemic spread rapidly northward, causing outbreaks of rotavirus-induced acute gastroenteritis in many communities spread over 1 million km^2^ in the northern Territory and in outback areas of southwestern Queensland and West Australia from May through July 2001 (Figure 1). The National Rotavirus Strain Surveillance Program received specimens from the hospitalized patients. Serotype analysis indicated that G9 strains were responsible for the outbreak (*11*).

**Figure 1:**
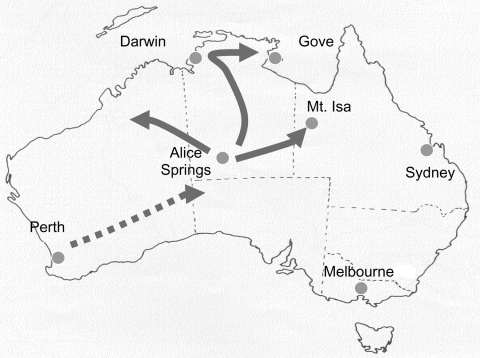
Map of Australia, indicating the locations where outbreaks of acute rotavirus gastroenteritis were identified during 2001. The direction of spread is indicated. Darwin-Alice Springs distance is 1,500 km.

This study describes the tracking and characterizing of serotype G9P[8] strains implicated in the outbreaks in central and northern Australia in 2001 and provides evidence that the outbreaks were caused by a single strain. The results highlight the importance of continued detailed epidemiologic and virologic studies of rotavirus serotypes that cause severe gastroenteritis.

## Materials and Methods

### Epidemiologic Features of Outbreaks

Alice Springs Hospital in Central Australia provides tertiary medical care for approximately 43,000 people living in a catchment area of >1 million km^2^. Approximately 28,000 people live in the city and 15,000 in small remote communities that range from 12 to 800 persons (*18*). Emergency transport to Alice Springs Hospital is provided when necessary by the Royal Flying Doctor Service. Each year epidemics of severe rotavirus diarrhea result in hospitalization of young children, usually during the cooler months from May to August. In May 2001, "one of the largest outbreaks of rotavirus in living memory swept through Central Australia," resulting in hospitalization of 137 children with confirmed rotavirus infection. Sixty-one percent of these children were from remote regions. More than 90% of the children were identified as aboriginal. Fifty-nine percent were <12 months of age, and 96% were <4 years of age (*18*). A much larger number of children were less severely affected. At one stage, the Alice Springs Hospital, the only hospital serving a scattered population of 55,000 people, had 74 of its 164 beds occupied by children with gastroenteritis. Extra nurses had to be flown 1,500 km from Darwin to assist (*18*).

The epidemic moved northward during the next 2 months (June and July), causing an increase in the number of children admitted to hospital with acute gastroenteritis in centers such as Darwin and Gove. These towns are >1,500 km from Alice Springs. In addition, cases of acute gastroenteritis were identified in remote communities to the northeast and northwest of Alice Springs, including Mount Isa (Figure 1).

### Stool Samples

During 2001, a total of 348 specimens, examined by enzyme immunosorbent assay (EIA) or heminested reverse transcription–polymerase chain reaction (RT-PCR) analysis, or both, were identified as serotype G9P[8]. Rotavirus G serotype was determined by using an in-house EIA assay that incorporates neutralizing monoclonal antibodies specific for G1, G2, G3, G4, and G9 antigens (*19**,**20*). The EIA was supplemented by RT-PCR analysis to determine G and P genotypes (*21**,**22*) The electropherotypes of all 348 G9P[8] strains were determined by polyacrylamide gel electrophoresis (PAGE) (*20*).

Fifteen G9P[8] rotavirus–positive specimens were selected for sequence analysis on the basis of electropherotype, location, and timing of sample collection (Table 1). Ten specimens were representative of strains from the 2001 outbreak and included six specimens from Alice Springs and the surrounding remote communities (Docker River, Hermannsberg, and Maryvale); Gove, Mount Isa, Darwin, and Perth were each represented by a single specimen. Five G9P[8] strains collected from children admitted to hospital in the cities of Alice Springs (1999), Perth (1999), Sydney (1999), and Melbourne (2000 and 2001) were used for comparison.

**Table 1:** Characterization of rotavirus strains recovered from infants in outbreak and nonoutbreak settings

Strains	Isolation location/date	Laboratory designation
Outbreak (2001)		
Ob-Perth-1	Perth 3/26/2001	3084375
Ob-AS-1	Alice Springs 5/12/2001	151572
Ob-Her-1	Hermannsberg 5/12/2001	151496
Ob-DR-1	Docker River 5/18/2001	152245
Ob-MV-1	Maryvale 5/23/2001	152433
Ob-AS-2	Alice Springs 5/25/2001	152704
Ob-AS-3	Alice Springs 5/30/2001	153004
Ob-Dar-1	Darwin 7/5/2001	6557 739
Ob-MI-1	Mt Isa 7/12/2001	6504 7767
Ob-Gv-1	Gove 8/7/2001	19522
Nonoutbreak		
MG9.06	Melbourne 9/1/2000	
Melb- G9.21	Melbourne 1/3/2001	
Syd-G9.1	Sydney 6/17/1999	991680093
Perth G9.1	Perth 9/20/1999	326924
AS-G9.1	Alice Springs 9/13/1999	8705

### RT-PCR Amplification

Rotavirus dsRNA was obtained from 10% fecal extracts by using a standard phenol-chloroform extraction and a hydroxyapatite purification method (*23*). The dsRNA gene segments, encoding proteins VP4, VP7, NSP1, and NSP4, were reverse transcribed and amplified by PCR.

Full-length gene 9 was amplified with primers Beg9 and End9 (*21*). For gene segment 4, primers con2 and con3 were used to amplify an 887-bp region encompassing the VP8 subunit of the VP4 gene (*22*). Gene segment 10 was amplified with primers complementary to the 3´ end of each RNA segment (*24*). A 400-bp fragment of the gene segment, encoding NSP1 protein, was amplified with internal primers (*25*).

### Sequence Analysis

PCR products were purified with gel extraction and spin column purification (Qiagen, Inc., Hilden, Germany). The nucleotide sequence of each PCR product was determined in both directions with the dideoxynucleotide chain terminator method with the BigDye sequencing kit (Perkin Elmer-Applied Biosystems, Foster City, CA) and specific oligonucleotide primers in an automated sequencer.

Sequences of each gene segment were analyzed with the Sequencher program (Gene Codes Corp., Inc., Ann Arbor, MI) and subsequently compared with other sequences by using E-CLUSTAL W and analyzed by using the DNAdist and Neighbor programs from PHYLIP software accessed through BioManager, Australian Genomic Information Service (ANGIS, University of Sydney). The statistical significance of the constructed phylogenies was analyzed with the Seqboot program to conduct bootstrap analysis, with 100 replicates (*26*). Phylogenetic trees were displayed with the Treeview program. The nucleotide sequences for genes encoding the VP7, VP8, NSP4, and NSP1 of the outbreak strains described in this study have been deposited in GenBank sequence databank and assigned the accession numbers AY629560–AY629562.

### Northern Hybridization Analysis

Northern hybridization analysis was carried out on four Australian G9 strains isolated during 2001, two outbreak strains from Alice Springs (Ob-AS -1 and Ob-AS-3) and two nonoutbreak strains from Melbourne (MG9.06 and Melb-G9.21). Northern hybridization was performed with whole genome probes derived from virus strains F45 (G9,P1A[8], SGII, Wa genogroup) and RV-5 (G2,P1B[4], SGI, DS1 genogroup). Probes were generated by labeling cDNA, derived by reverse transcription of purified dsRNA with random hexanucleotide primers and digoxigenin (DIG)-11-dUTP. Northern hybridization was performed with 10 ng/mL of probe and performed under stringent conditions (*24*).

## Results

### Epidemiologic Features of Rotavirus Outbreak

**Figure 2:**
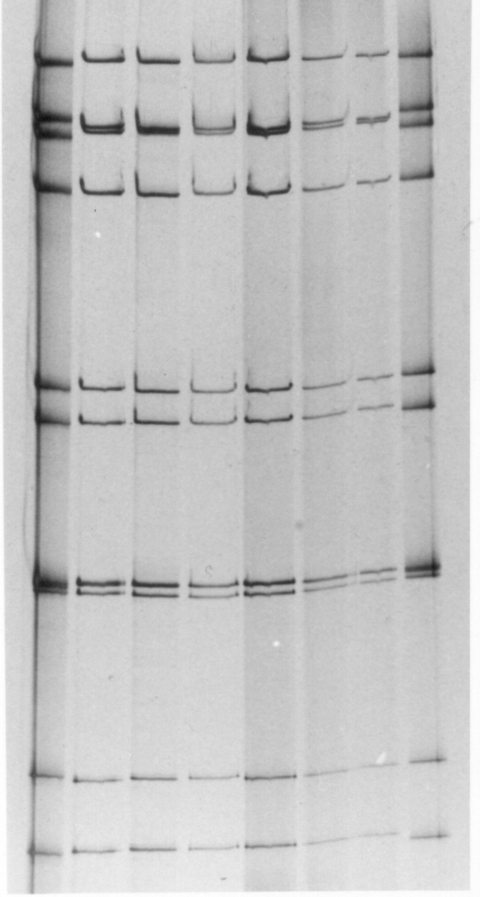
Electrophoretic patterns of the dsRNA of G9P[8] rotavirus strains obtained from Australian children with acute gastroenteritis during the rotavirus outbreak, 2001. Electropherotypes of four representative G9P[8] strains isolated from children during the 2001 rotavirus outbreak are illustrated in part A. Lane 1, Alice Springs (Ob-AS-1); lane 2, Darwin (Ob-Dar-1); lane 3, Gove (Ob-Gv-1); and lane 4, Mount Isa (Ob-Gv-1). B) compares the electropherotypes of G9P[8] strains isolated from children prior to the 2001 rotavirus outbreak with a strain isolated in Alice Springs during the 2001 outbreak. Lanes 1 and 8; Ob-AS-1, Lane 2; Alice Springs 1999, lane 3; Sydney 1999, lane 4; Perth 1999, lane 5; Melbourne 1999, lane 6; Melbourne 2000, lane 7; Melbourne 2001.

Serotype analysis of specimens obtained from children hospitalized during the gastroenteritis epidemic showed that rotavirus serotype G9 was the predominant type identified during the Central and northern Australian 2001 outbreak (Table 2) (*17*). Serotype G9 strains were the most commonly identified 69% (111/161) in Alice Springs, 81% (60/74) in Darwin, and 100% (31/31 and 25/25) in Gove and Mount Isa. Serotype G9 represented 25.2% of specimens from Western Australia. However, the results from Western Australia include samples collected from two regions, one urban (Perth) and the other remote communities in the northwestern Australian outback. G9 represented 18.6% in the urban collection and 45.1% in the remote collection. All samples obtained during the epidemic outbreak (Alice Springs, Darwin, Gove, Mount Isa, and Western Australia) had an identical long electropherotype (Figure 2A), which was distinct from the patterns of serotype G9 strains identified in other Australian locations (Melbourne, Sydney, Perth, and Alice Springs) during previous rotavirus seasons (Figure 2B).

**Table 2:** Serotyping results from Australian centers January–December, 2001

Location	n	% of rotavirus-positive samples by serotype					
		G1	G2	G3	G4	G9	NT^a^
Western Australia							
Urban	306	67.5	0.3	0.3	–	18.6	15.1
Remote	102	34.3	1	–	1	45.1	18.9
Northern Australia (including Mt. Isa)							
Alice Springs	161	24.8	–	–	–	69	6.2
Darwin	74	5.4	–	–	1	81	12.2
Gove	31	–	–	–	–	100	–
Mt. Isa	25	–	–	–	–	100	–
Southern Australia							
Melbourne	176	48.3	4.6	0	6.8	10.2	30.1

^a^Nontypeable results include samples with mixed serotype results and samples that do not react with any of the serotyping monoclonal antibodies.

### Sequence Analysis

**Figure 3:**
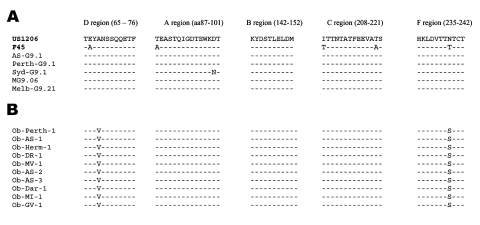
Deduced amino acid sequences of the VP7 antigenic regions of rotavirus G9P[8] strains. A) Outbreak strains. B) Nonoutbreak strains. The VP7 sequences of the standard G9 strain (US1206) and the Australian nonoutbreak strains were obtained from GenBank. Accession numbers are as indicated: US1206: AJ250271, Perth G9.1: AY307094, Syd-G9.1: AY307093, MG9.06: AY307085, Melb-G9.21: AY307090. The sequence of rotavirus strains F45 was obtained from Kirkwood et al. (*33*). All other sequences were determined in this study. A dash indicates homology with the US1206 sequence at that position.

The gene encoding VP7 was sequenced for nine representative strains isolated from several locations during the outbreak (Ob-Perth-1, Ob-AS-1, Ob-Her-1, Ob-DR-1, Ob-Mv-1, Ob-AS-3, Ob-Dar-1, Ob-MI-1, and Ob-Gv-1). The VP7 genes were highly conserved, and all outbreak strains were identical at both the nucleotide and amino acid level. Comparison of the VP7 gene between the outbreak strains and other serotype G9 strains isolated from 1999 to 2000 in Melbourne before the outbreak (MG9.06, Melb-G9.21, and Syd-G9.1), Perth (Perth G9.1) and Alice Springs (AS-G9.1), indicated a highly conserved gene. The VP7 genes had nucleotide and amino acid homology of >99% identity. Alignment of the deduced amino acid sequences of the VP7 gene showed three conserved amino acid substitutions between the outbreak and nonoutbreak strains (Figure 3). These were at position 242 (Asn–Ser) in the antigenic F region, at position 68 (Ala–Val) in antigenic region D and at amino acid position 40 (Leu–Phe), which is outside of the major antigenic regions.

The nucleotide sequences of the VP8 subunit of the VP4 gene were determined and compared for three outbreak strains (Ob-Perth-1, Ob-AS-1, and Ob-AS-2) and two nonoutbreak strains (MG9.06, Melb-G9.21). Analysis of the predicted amino acid sequence indicated that the P[8] VP4 gene of all Australian serotype G9 strains (outbreak and nonoutbreak) had an F45-like P sublineage; 9 of the 11 amino acid positions used to classify the P sublineages were conserved with the F45-like residues (*27*). At position 162, all of the Australian strains possessed the Wa-lineage residue of arginine, and at position 195, a Gly was observed rather than Asp or Asn. VP8 genes of the outbreak and nonoutbreak strains differed. Comparison of the amino acid sequences of the outbreak and nonoutbreak strains showed three conserved differences at positions 21 (Glu–Lys), 91 (Ile–Val), and 249 (Val–Ile).

The complete gene segment 10 sequence, encoding the NSP4 protein, was determined for four outbreak strains (Ob-Perth-1, Ob-AS-1, Ob-AS-3, and Ob-Dar-1) and two nonoutbreak strains (MG9.06, Melb-G9.21). All of the outbreak strains had identical nucleotide and deduced amino acid sequences and at least 97% nucleotide identity and amino acid homology with the nonoutbreak strains. Alignment of the deduced amino acid sequences showed five conserved differences, at aa53 (Thr–Ala), aa76 (Val–Ile), aa141 (Thr–Ile), aa142 (Ile–Val), and aa161 (Asn–Ser) between the outbreak and nonoutbreak strains.

### Northern Hybridization Analysis

Northern hybridization analysis was conducted on the Australian serotype G9 strains to investigate the genomic relationship among the gene segments from the outbreak and nonoutbreak strains (data not shown). Hybridization results showed that 10 gene segments hybridized strongly with the F45 whole genome probe, indicating that the Australian G9 strains belong to the Wa genogroup. Gene segment 5 of F45 failed to hybridize in all the outbreak and nonoutbreak strains. Partial nucleotide sequence analysis of the gene segment 5 (215-620 nt) NSP1 was conducted. A GenBank search showed that this gene had greatest identity (95%) with gene 5 from the human neonatal strain ST-3. Alignment of the deduced amino acid sequences between the outbreak strains (Ob-AS-1 and Ob-AS-3) and nonoutbreak strains (MG9.06 and Melb-G9.21) showed a highly conserved region with no conserved amino acid substitutions.

### Tracking Rotavirus Outbreak G9P[8] Strain

**Figure 4:**
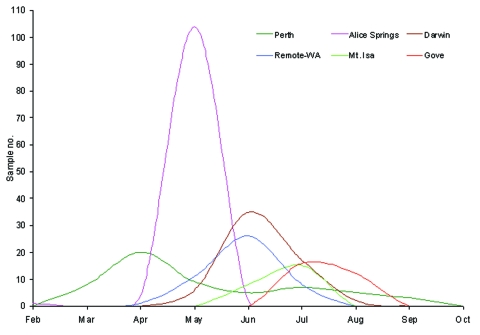
Temporal appearance of rotavirus G9P[8] strains isolated from children admitted to hospital with acute gastroenteritis during 2001. The monthly appearance of G9P[8] strains for each of the collaborating laboratories is indicated. The Western Australia results are divided in urban location (Perth) and remote outback locations (remote-WA).

The temporal appearance of rotavirus G9 infections in each of the geographic regions studied is illustrated in Figure 4. On the basis of these results, the suggested direction of spread of this strain is indicated in Figure 1. The rotavirus outbreak G9 strain was identified first in Perth, Western Australia, in March/April, and then in Central Australia, where the peak prevalence of rotavirus diarrhea occurred in Alice Springs in May. The outbreak strain then spread rapidly northward to Darwin in June and July. Outbreaks to the west (Docker River) and northeast (Mount Isa, Gove) of Alice Springs were also identified during June and July 2001.

## Discussion

We report the genetic characterization of rotavirus G9P[8] strains isolated during the outbreak of severe rotavirus diarrhea that occurred in Central Australia during 2001. Results described in this study comprising PAGE analysis of all the outbreak strains and sequence analysis of gene segments encoding VP7, VP8, and NSP4 from representative strains illustrate that the Central Australian rotavirus outbreak was the result of the spread of a single strain of serotype G9. This strain was distinct from serotype G9 strains present in Melbourne during the same year (2001) and in previous rotavirus seasons in Alice Springs and other Australian locations, including Melbourne, Sydney, and Perth (*28*).

Serotype G9 is the most widespread of the emerging rotavirus serotypes. Recent epidemiologic studies suggest that this type represents a common global serotype. In Australia, serotype G9 has progressed from the initial identification of three isolates in 1997 (*10*) to the second most common serotype from 1999 through 2001, to becoming the most common serotype in Australia during 2002 (40% of all isolates) and 2003 (74%) (*11**,**17*). Similarly, serotype G9 was identified as the prevailing serotype in several Japanese cities from 1998 through 2000 (*29*).

This outbreak represented one of the largest outbreaks of rotavirus disease in Central Australian history. The outbreak, strain while appearing to have its origins in urban Perth, on the coast of Western Australia, had its major effect on remote communities in Central Australia. During May 2001, a total of 246 children with acute gastroenteritis arrived at the emergency department of the Alice Springs Hospital; 137 children were hospitalized. The outbreak stretched medical and staffing resources to capacity (*18*). In Alice Springs, serotype G9 represented 69% of the typeable strains. The predominance of serotype G9 increased with the northwards spread of the outbreak. The tracking of this outbreak was possible because the timing of the rotavirus activity varied by geographic location, with each community being discrete and remote from the others. This finding was highlighted in Darwin and Gove, where 81% and 100% of the isolates were identified as serotype G9, respectively. We have shown that the Central Australian outbreak strain appeared to originate from serotype G9 strains present in Perth, Western Australia, during early 2001. The Perth G9P[8] strains possessed an identical electropherotype and identical gene sequences encoding for VP7, VP8, and NSP4 proteins to strains isolated earlier during the Central Australian outbreak.

The emergence and spread of the G9 "outbreak" strain corresponded with several deduced amino acid changes in viral proteins VP8, VP7, and NSP4 compared with nonoutbreak strains from Melbourne, Sydney, Perth, and Alice Springs. These proteins have previously been shown to influence virulence of rotavirus strains (*30**,**31*). Conserved changes in the gene coding for VP7 were identified in two important antigenic regions (regions D and F). These genetic changes likely affect the function of the VP7 protein virulence. One change is adjacent to the glycosylation site Asn-X-Thr at residues 69–71, a site common to all human rotavirus strains. The substitution at position 68 (Ala–Val) may influence glycosylation and hence alter the antigenic reactivity of this virus. An effect of glycosylation on virus antigenicity and virulence has been previously postulated (*30**,**32*). Additional evidence that the removal or addition of N-linked carbohydrates can influence viral antigenicity comes from several studies using N-MAbs and polyclonal antiserum (*32**,**33*). The conserved substitution identified in the antigenic F region of the VP7 protein may also be important in virulence. This antigenic region has previously been shown to contain neutralization epitopes of serotype G9 viruses, and the region was only accessible in viruses that lacked glycosylation site in this region such as serotype G9 (*33*). This region may represent an immunodominant region in G9 viruses. The alterations in this region identified in this study may have altered the antigenicity of the strains such that they were able to avoid immune detection. This scenario has been used to explain the reemergence of rotavirus serotypes, in particular serotype G2, and the resultant intermittent epidemics. Alterations in the antigenicity of rotavirus serotype G2 strains were conferred by amino acid substitutions in the antigenic A region of the VP7 protein. A higher incidence of infection with these strains occurred in older children in the United Kingdom from 1995 through 1999, which suggests that cross-protective antibody failed to afford protection against these serotype G2 strains (*34*).

NSP4 has been shown to act as an enterotoxin in mice and is involved in virus pathogenesis by acting as a receptor for double-layered particles (*35*). Several studies have found that NSP4 has been associated with altered virulence, by sequence comparison of symptomatic and asymptomatic strains isolated from serotypically identical human strains (*30**,**31*) or symptomatic and asymptomatic porcine strains tested in a mouse model (*36*), or by gene reassortment studies in a piglet model (*37*). Our study has identified changes in important regions of this protein of the outbreak strain. Specifically, changes have been identified in a region of NSP4 critical for VP4 binding (aa112–148) and in a region associated with membrane destabilization (aa48–91) (*38*). These changes may affect virus stability. NSP4 has been shown to elicit an immune response in humans (*39*). However, the influence of these changes identified on immune recognition is unknown, since the antigenic regions are uncharacterized.

The electropherotype of the G9P[8] outbreak strains differed in the several respects from G9P[8] strains identified elsewhere in Australia from 1999 to 2001. Differences were most apparent in the mobility of gene segments 2 and 3. As yet, no evidence shows that genes 2 and 3 (coding for VP2 and VP3, respectively) are involved in virulence (*37*).

Northern hybridization analysis of both outbreak and nonoutbreak serotype G9 strains had a similar hybridization pattern. Ten of 11 segments hybridized to a Wa-like probe, which indicated that these strains all belong to the human Wa genogroup. Only gene segment 5, which encodes the NSP1 protein, failed to hybridize. The gene 5 showed greatest identity to gene 5 of the P[6] strains ST3 and M37, rather than P[8] strains. Several studies have shown that the G9 VP7 protein is capable of associating with both VP4 proteins P[6] and P[8], in addition to VP6 proteins from subgroup I and II (*3**,**16**,**40*). A previous study has associated gene 5 with pathogenicity in the mouse model (*41*). However, alterations in the gene 5 segment cannot explain the emergence of this strain in Central Australia since limited sequence analysis failed to identify any genetic difference between the outbreak and nonoutbreak G9 strains. The results from this study further highlight the ability of serotype G9 strains to undergo reassortment and extend this observation to include gene segments that encode non-structural proteins.

Rotavirus surveillance programs using molecular assays have shown that most cases of acute gastroenteritis are associated with the globally common serotypes, G1–G4. However, the emergence of novel or rare serotypes, including the identification of serotypes G5, G6, G8, and G10, in children in many settings worldwide, has highlighted a much greater strain diversity than previously reported. The diversity of rotavirus strains has arisen because of the strains ability to undergo genetic evolution. A number of different mechanisms exist by which rotavirus strains can evolve, ranging from reassortment of single or multiple gene segments during mixed infections by strains of human-human origin or human-animal origin to generation of single point mutations in immunologically important genes. In particular, serotype G9 strains appear to have an enhanced capacity to reassort. Epidemiologic studies have identified G9 strains in combination with several different VP4 genogroups (P[4], P[6], P[8], and P[11]) and with both VP6 subgroup antigens (*3**,**9**,**12**,**24**,**28**,**40*). These mechanisms provide rotavirus with a unique capacity to rapidly evolve, and produce strains that have the potential to be epidemiologically important. Protection from rotavirus disease relies on production of heterotypic immune responses after primary infection. However, novel strains may avoid stimulating preexisting immunity produced from previous rotavirus infections because of the unique nature of the outer capsid proteins. Outbreaks of acute gastroenteritis associated with an unusual serotype G2 strain and serotype G9 are two recent examples from Central Australia (*42*). Therefore, the diversity of rotavirus serotypes has important implications for vaccine development, especially if strains that are not targeted by current vaccine candidates continue to emerge as common types either globally or regionally.

National surveillance data since 2001 highlights the continued emergence of serotype G9 as the most prevalent serotype nationally, which results in the replacement of serotype G1 as the dominant strain for the first time since Australian rotavirus surveillance began in 1993. This study, which shows that a single rotavirus serotype G9 strain was responsible for a large epidemic of severe gastroenteritis in Central Australia, emphasizes that sequence alterations on viral proteins may be implicated in virus virulence. Continued surveillance of rotavirus serotypes, which includes the capacity to identify new and emerging serotypes, is important for successfully developing and implementing vaccines.
